# A Model for Basic Emotions Using Observations of Behavior in *Drosophila*

**DOI:** 10.3389/fpsyg.2019.00781

**Published:** 2019-04-24

**Authors:** Simeng Gu, Fushun Wang, Nitesh P. Patel, James A. Bourgeois, Jason H. Huang

**Affiliations:** ^1^Institute of Brain and Psychological Science, Sichuan Normal University, Chengdu, China; ^2^Department of Psychology, Jiangsu University, Zhenjiang, China; ^3^College of Medicine, Texas A&M University, College Station, TX, United States; ^4^Department of Psychiatry, Baylor Scott & White Health, Dallas, TX, United States

**Keywords:** basic emotions, core affection, monoamine, evolution, instinct, emotion flow, *Drosophila*

## Abstract

Emotion plays a crucial role, both in general human experience and in psychiatric illnesses. Despite the importance of emotion, the relative lack of objective methodologies to scientifically studying emotional phenomena limits our current understanding and thereby calls for the development of novel methodologies, such us the study of illustrative animal models. Analysis of *Drosophila* and other insects has unlocked new opportunities to elucidate the behavioral phenotypes of fundamentally emotional phenomena. Here we propose an integrative model of basic emotions based on observations of this animal model. The basic emotions are internal states that are modulated by neuromodulators, and these internal states are externally expressed as certain stereotypical behaviors, such as instinct, which is proposed as ancient mechanisms of survival. There are four kinds of basic emotions: happiness, sadness, fear, and anger, which are differentially associated with three core affects: reward (happiness), punishment (sadness), and stress (fear and anger). These core affects are analogous to the three primary colors (red, yellow, and blue) in that they are combined in various proportions to result in more complex “higher order” emotions, such as love and aesthetic emotion. We refer to our proposed model of emotions as called the “*Three Primary Color Model of Basic Emotions*.”

## Introduction

Emotions are fundamental to human life (Kvajo, 2016); when expressed pathologically, psychiatric disorders of emotional regulation, such as depressive and bipolar disorders, are leading causes of medical disability. Despite the importance of emotion in human health and illness, scientists struggle to reach consensus on the constructs underlying emotional phenomena and experiences (LeDoux, 1995; LeDoux J., 2012). In addition, controversy abounds over the definitions of emotion, the number of discrete, fundamental emotional states that exist, and the degree to which different emotions have distinct neurophysiological signatures (LeDoux, 1995; Damasio, 1997). Insects, such as *Drosophila*, offer animal models for studying the mechanisms of fundamental emotional processes (Anderson and Adolphs, 2014; Hoopfer, 2016; Shpigler et al., 2017). It is important to use the powerful analytical tools available in invertebrate model organisms to understand the evolutionary origins and neurobiological underpinnings of emotions. Darwin (1876) found that insects use certain behaviors to express feelings homologous to human emotions, such as stridulation, which is the act of creating sounds by rubbing certain body parts together and can represent a range of various emotions in some insects. Darwin (1876) assumed that emotion-associated behavioral phenotypes are easily recognizable in many species, including insects (Anderson and Adolphs, 2014).

We have developed a theory of primary emotions using behavioral observations of *Drosophila*. Basic emotions are internal states induced by basical bodily changes, and can in turn induce genetically “hardwired” instinctual behaviors. They are highly conserved throughout evolution, and exhibit certain functional and adaptive properties that are shared across a wide phylogenetic range. For example, emotions such as fear and anger are thought to have evolved in response to fundamental life challenges and threats. Anderson and Adolphs (2014) suggested that these primary emotions (when combined) provide a framework for creating various types of secondary emotions, such that elements of primary emotions can be combined with the experience of other, higher order emotions that are more affected by specific learning and experience. Using this approach, primary emotions are observable in evolutionarily diverse organisms, allowing us to functionally “dissect” the mechanisms of the presumed associated internal emotional states and their externally manifest behaviors. There are many reports associating presumed fear and anger emotions with “fight or flight” behaviors in *Drosophila* (Kravitz and Fernandez, 2015). From analysis of these basic emotions and their associated behavioral phenotypes in animal models, we elucidate mechanisms of basic emotions in humans and propose to utilize this insight to define the mechanisms of disorders of emotional regulation.

## Emotion Theories

During the last century, the two most widely accepted theories in affect studies are basic emotion theory and dimensional theory. However, these two theories have been contradictory to each other, and have been described as being in a “100 years war” against each other (Lindquist et al., 2013; Barrett and Russell, 2015). The difference lies in whether emotions are characterized as discrete entities or an independent dimension (Bestelmeyer et al., 2017). Here we give an integrative theory in which we propose that these two theories not necessarily contradictory.

### Basic Emotion Theory

Basic emotion theory has been very influential for more than half a century, providing inspiration for interventions in psychopathology (Saarimaki et al., 2016; Celeghin et al., 2017; Williams, 2017; Hutto et al., 2018; Song and Hakoda, 2018; Vetter et al., 2018; Wang et al., 2018). Theories about basic emotions originated from ancient Greece and China (Russell, 2003). Current basic emotion theory started with Darwin (1872) and Ekman (2003), and later (Tomkins, 1962), subsequently followed by Ekman (1984), and Izard (1977), then by many current psychologists (Ortony and Turner, 1990; Panksepp, 2007; Scarantino and Griffiths, 2011; Gu et al., 2016, 2018; Saarimaki et al., 2016; Hutto et al., 2018). Basic emotion theory proposes that human beings have a limited number of emotions (e.g., fear, anger, joy, sadness) that are biologically and psychologically “basic” (Wilson-Mendenhall et al., 2013), each manifested in an organized recurring pattern of associated behavioral components (Ekman, 1992a; Russell, 2006). Izard (1977) argued that the basic emotions are preserved because their biological and social functions are essential in evolution and adaption; he further suggested that basic emotions have innate neural substrates and universal behavioral phenotypes (Shpigler et al., 2017). In a special issue of *Emotion Review*, several research psychologists outlined the latest thinking about each theoretical model of basic emotions (Plutchik, 1962; Ekman and Friesen, 1969; Ekman, 2003; Izard, 2010, 2011; Ekman and Cordaro, 2011; Levenson, 2011; Panksepp and Watt, 2011; Tracy and Randles, 2011).

Basic emotions evolved to handle fundamental life tasks, e.g., fear and anger can aid survival by influencing an organism to either flee for safety or fight to defend itself. The elements of basic emotions can be combined to form complex or compound emotions (Ekman, 1992b). Even though many psychologists have accepted the theory of basic emotions, there is no consensus about the precise number of basic emotions. Robert Plutchik proposed eight primary emotions: anger, fear, sadness, disgust, surprise, anticipation, trust and joy, and arranged them in a color wheel. Ekman proposed seven basic emotions: fear, anger, joy, sad, contempt, disgust, and surprise; but he changed to six basic emotions: fear, anger, joy, sadness, disgust, and surprise. However, a recent study found that disgust and anger shared similar wrinkled nose, and fear and surprise shared raised eyebrows (Jack et al., 2014). The differences between anger and disgust and the differences between fear and surprise, are thought to have developed later for social functions and not for survival *per se* (Mansourian et al., 2016). As such, Jack et al. (2014) proposed that we humans have four basic emotions: fear, anger, joy, and sad. Notably, other authors have also proposed fear, anger, joy, and sadness as four basic emotions (Gu et al., 2015, 2016; Wang and Pereira, 2016; Zheng et al., 2016). As Izard said: people need the category label of *fear* to explain flight to one another for safety, *anger* to explain the frustration of blocked goal responses, *joy* (or its equivalent) to explain the pride of achievement, and *sadness* to explain the experience of a life-changing loss (Izard, 2007).

### Dimensional Theory of Emotion

**FIGURE 1:**
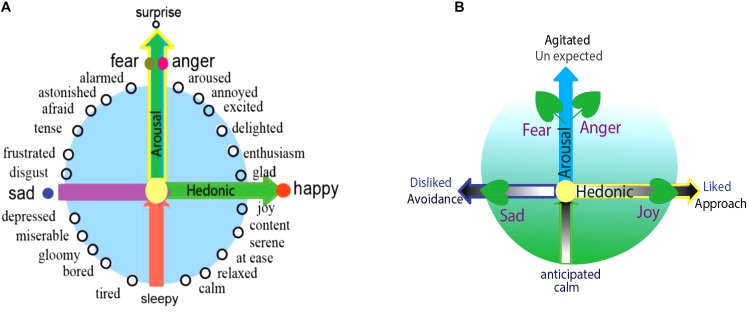
Core affects and basic emotions. **(A)** All emotions, including the basic emotions, can find their locations in the circle of the Circumplex, which means that different emotions have different *arousal or hedonic parameters*. The reason for basic emotions being “basic” is that they can also be arranged on the special location in a two-dimension coordinate plane. And the typical locations of the four basic emotions suggest that they have different parameters (core affects): happiness and sadness are due to the hedonic value of the stimulus (physiological needs), while the fear and anger depend on the way the stimulus occurs (safety needs). **(B)** The locations of basic emotions in the dimensions are also decided by the stimulus that induce them, and also in the behavioral responses. The stimulus has two features: whether it fits into our needs (hedonic value) or whether it happens expected (arousal). The behavioral responses the emotion induced has two features: approach (hedonic value) and agitation (arousal value).

Dimensional studies of emotions originated from Wundt (1897), later followed by Scholsberg (1954), who proposed that emotions can be defined by three-independent dimensions: pleasant-unpleasant, tension-relaxation, and excitation-calm. Later, many others found that the last two dimensions are actually overlapping. Ekman (1957) also proposed a pleasant-unpleasant and active-passive scale as sufficient to capture the difference among emotions. Then Russell’s (1980) invented the circumplex, and proposed that all emotions can be arranged in a circle controlled by two independent dimensions: hedonic (pleasure-displeasure) and arousal (rest-activated) (Figure 1, left) (Russell, 1980; Russell and Barrett, 1999; Posner et al., 2005; Barrett and Russell, 2015). The horizontal axis of the circumplex is hedonic and the vertical axis is arousal; accordingly, the different location of each emotion on the quadrant reflects varying amounts of hedonic and arousal properties (Figure 1; Posner et al., 2005).

### Integration of Basic Emotion Theory and Dimensional Theory

Basic emotion approach differs from the dimensional approach in that the latter suggests that emotions are fundamentally the same, only differing in intensity or pleasantness (Ekman, 2003), while the former proposes that emotions are composed of limited number of basic emotions. Here we propose an integrative approach wherein basic emotions also differs in the intensity or pleasantness, like all other emotion. Therefore, basic emotion theory is not contradictory to dimensional theory. The dimensional approach proposes that every emotion has different amounts of hedonic and arousal value. The hedonic (pleasure-displeasure) and arousal (rest-activated) value, which can be called core affects (Russell, 2003; Barrett et al., 2007), are essential features of all emotions, including basic emotions (Gu et al., 2016). Therefore, basic emotions, like all other emotions can find their location in the circumplex.

The specificity of basic emotions on the circumplex is that they located on the axis of the dimensions, which might be the reason that they are “basic.” Happiness and sadness are on the opposite sides of the horizontal dimension, implying that they are a reflection of the hedonic value of the stimulus and unrelated to the safety value (Figure 1, left). Fear and anger are on the vertical axis, implying they are based on the safety value of the stimulus and independent upon the hedonic value of the stimulus. Because of their special location, the basic emotions (fear or anger) have “0” amounts of hedonic value, and the basic emotion of joy or sadness has “0” amounts of safety value. Therefore, the reason for basic emotions being “special” lies in that they only represent only one core affect, because of their specific location. Thus, we introduce a prerequisite conditions for basic emotions: *Basic emotions should locate on the axis of the two emotional dimensions*. Emotions located on the axis of the dimensions are basic emotions, which might suggest that we have four basic emotions: fear-anger, joy, and sadness. Complex emotions can also find their locations on the quadrant, such as love or aesthetic emotions.

### Factors Affecting the Locations of Emotion in the Dimensional Plane

Ekman (1992b) said all emotions differ in the stimulation events, appraisals, behavioral response, and physiological responses. The locations of all emotions on the dimensions can be defined by these factors. According to the appraisal theory, emotions are internal states which are activated by stimulation events. Every stimulation event has two features: whether it is fit for our needs (hedonic value), and whether it happens as expected (arousal) (Figure 1, right). These two features correspond to the two core affects: the hedonic value that represents a physiological need, and the safety value represents the way the stimulus happens (Wang and Pereira, 2016; Zheng et al., 2016). The two-dimensional coordinate plane or the core affects also represents these values of the stimulus: whether it happens as expected, and whether it is fit for our needs (Gu et al., 2016). Lazarus distinguished two kinds of appraisals at a stressful stimulation (Figure 1, right) (Izard, 1977). The first is automatic, unconscious, fast-activating, is related to harm and threat, and induces fearful emotion to motivate avoidance and withdrawal, whereas the second appraisal (he named reappraisal) is conscious and associated with coping (Lazarus, 1999; Zheng et al., 2016). Fear and anger both result from unexpected stressful events, and while fear is associated with feelings of uncertainty, anger is associated with planning to cope with the stressful situation (Figure 1, right) (Moons et al., 2010). Ultimately, fear and anger depend on the manner the stimulation event occurs (Gu et al., 2016), or fear and anger are “twin” emotions, they are two sides of one coin (Gu et al., 2015), and they locate on the top of the vertical dimension.

The locations of emotions on the dimensional plane are also predicated by the behaviors they might induce. Emotion is an internal state, not a behavior (Baker, 2004). Emotion is a tendency of behavior (Roseman, 1984), because the emotion can be separated from the behavioral actions it induces. For example, we can block the actions associated with angry emotion. However, the emotion induced behaviors have two features: the direction and the tension of the behavior (LeDoux, 1998; LeDoux and Brown, 2017). These features can be reflected on the dimensions: hedonic value decides the approach/avoidance, the vertical dimensions decides the tension of the behavior (Wang and Pereira, 2016; Zheng et al., 2016). Thus, the locations of the emotions on the dimension can also be determined by the behaviors they might induce. Fear and anger can induce “flight or fight,” which have opposite directions: fear is in the negative direction (Certel et al., 2010), while fight is in the positive direction. Analogously, joy and sadness can induce approaching or avoidance, respectively (Arnott and Elwood, 2009).

Therefore, like what Lazard suggested, in face of a stressful situation, the individual will collect energy to cope with the situation with “fear or anger” emotions, and induce “fight or flight” actions. After coping with the situation, the individual will have an opportunity for reappraisal, which Lazarus named as emotion-focused coping (Izard, 1977). If the individual have successfully coped with the stressful situation, he/she will feel happy; otherwise, he will feel sad (Aldwin, 1994; Lazarus, 1999). Therefore, Frijida said, sadness is acceptance of the failure, without more efforts to fight (Frijda, 1986). In all, the two dimensions of emotions not only represent the two different feature of a stimulus (hedonic value and safety value), but also represent two features of the behaviors they induced: direction (approach or avoidance) and activation.

## Evolutionary Aspects of Basic Emotion

Human emotions are poorly understood even though there is a long venerable tradition of research directly at understanding them. One problem is that physiologists and psychologists who study emotions do not necessarily look at emotion enough from an evolutionary standpoint (Ramachandran and Jalal, 2017). This is unfortunate because as Dobzhansky famously said, “Nothing in biology makes any sense except in the light of evolution” (Ramachandran and Jalal, 2017). However, comparative psychologists have also struggled with the problem of emotion because emotions cannot be directly observed or measured, and we have no access to the animals’ subjective experiences (Ramachandran and Jalal, 2017). Human being and some other animals (such as primates) can consciously know the subjective feelings (Kamitani and Tong, 2005; Kuppens et al., 2013); but other animals, who have no power of self-observation and cannot consciously know their internal states; they still have emotions (Perlovsky, 2016a). For the invertebrate animals, an emotion is just an internal state, which includes the need, drive, or tendency to a kind of behavior (Mesquita and Frijda, 2011).

We can only study emotions in the invertebrate animals depending on the animal’s behavior and physiology-emotional expression (Cosmides and John, 1995). Darwin (1872) was first to study the animal behaviors for emotions, and he suggested that even insects use certain behaviors to express feelings homologous to human emotions. In order to survive, animals need to sense their environment, evaluate the surrounding stimuli with their internal needs, and initiate appropriate behavioral responses (Kaun and Rothenfluh, 2017). Evolutionarily, emotional behaviors should first have direct survival value that was honed by natural selection, and these behaviors are adaptive responses to the environment that increase the chances of survival (John and Leda, 1990; Cosmides and John, 1995). The basic emotions were selected through evolution in order to promote the survivability of the species in their specific primitive environment (LeDoux J.E., 2012). Therefore, the basic emotion related behaviors are the naturally born instinct behaviors, which are evolutionarily adaptive (Ramachandran and Jalal, 2017). In addition, these basic emotion related behaviors are manifested as stereotypical behavioral phenotypes.

### Basic Emotions Are Related to Instincts

Basic emotions are thought to be universal as they are related to the most basic needs of the body, or the bodily instinctual needs (Schoeller et al., 2018). These few emotions constitute the most ancient and noticeable emotions, while other emotions (complex emotions), such as aesthetic emotions, are related to higher needs of our human experiences (Perlovsky, 2012). Grossberg and Levine, 1987 proposed a famous theory about instinct, which considers that the instinct resembles “neural sensors that measure vital parameters important for survival” (Grossberg et al., 1987; Perlovsky, 2016c), “for example, a low blood glucose level specifies an instinctual need for food” (Schoeller et al., 2018). Therefore measurement of glucose level is a kind of neural sensor. Grossberg and Levine theory proposed that emotions are the neural signals that connect instinctual sensor with the conscious brain, to make the instinctual needs to be consciously known to the brain, to make the instinct conceptually recognized-understood (Fontanari et al., 2012). William James also proposed that feelings are derived from sensing the body states (Damasio and Carvalho, 2013). Therefore, emotion is a kind of internal neural activity, whose major function is to sense the bodily needs, and then motivates behaviors depending on the external stimulus (Schoeller et al., 2018). Therefore basic emotions indicate satisfaction of instincts (Fontanari et al., 2012).

Remarkably, studies in *Drosophila* confirmed these hypothesis. Recent studies reveal that a small number of specialized central brain neurons in *Drosophila* brain directly sense specific circulating macronutrients (Pool et al., 2014), monitor systemic energy balance and alter feeding probability based on internal nutritional state (Pool and Scott, 2014). In addition to internal nutrient sensors, the flies can distinguish sugars based solely on caloric content in the absence of sweet taste detection (Dus et al., 2011). Furthermore, there is accumulating evidence that all animals have the central sensors to directly detect the levels of circulating carbohydrates and amino acids (Hao et al., 2005; Domingos et al., 2013). These studies thus identify central brain mechanisms that sense availability of specific nutrients, convert it to a change in neuromodulator output, and promote or inhibit feeding (Pool and Scott, 2014).

The basic emotions are evoked by sensing the basic bodily instinctual needs, while the “complex” emotions, including love, aesthetic emotions are evoked by higher cognitive needs of human being. In Maslow’s Hierarchy of Needs, physiological needs and safety needs might be directly related to basic emotions, while the other needs, such as the need for love, esteem and self-actualization, are related to feelings such as aesthetic emotions (Zheng et al., 2016). Kant (1951) said aesthetic emotions are related to need of knowledge in human being. Perlovsky also proposed that human beings have the special need for knowledge, which was named “knowledge instinct.” Aesthetic emotions are related to the knowledge needs, are related to learning, or understanding, and they are shown in many fields, such as mathematics, music, or even language (Perlovsky, 2015; Schoeller and Perlovsky, 2016). Aesthetic emotions can also be seen in creativity, and they are also related the NE and DA neuromodulator (Gu et al., 2018). *In all, the emotions are evoked by these sensing for bodily needs, and underlined by the neuromodulator release, and will promote some behaviors*.

### Basic Emotions Are Primitive

Basic emotions are basic due to the fact that they fit for primary life needs, and are developed early phylogenetically and ontogenetically (Ekman, 2003). The idea that invertebrates exhibit basic forms of emotions is increasingly accepted (Arnott and Elwood, 2009). Animals spend a significant amount of time seeking and selecting food for eat, while striving to avoid being eaten (John and Leda, 1990; Cosmides and John, 1995). These two forms of needs, which can be called hedonic and safety values, are two equally important, independent needs (Zheng et al., 2016). In Maslow’s Hierarchy of Needs, physiological needs were proposed to be superior to safety needs, with the idea that safety needs emerge only once physiological needs are satisfied (Maslow, 1948). However, an animal will not eat in a dangerous situation since the primary objective in such situations is assurance of safety (Zheng et al., 2016). Wild animals navigating the rich environments will face complicated situations with many uncertainties; accordingly, the evolution of an adaptive mechanism to first perform a safety check of the surroundings is critically important for its survival (Gu et al., 2016; Zheng et al., 2016). Maslow (1948) also said that “practically everything looks less important than safety, even sometimes the physiological needs which being satisfied, are now underestimated. A man may be characterized as living almost for safety alone” (Maslow, 1948). Therefore, in our previous paper, we proposed that safety needs might be more basic (Zheng et al., 2016). Anyway, *physiological needs and safety needs represent two equally important dimensions of human needs, which are independent from each other*. Darwin said “the fear emotion does not depend on experiences; instead it depends absolutely on heritage.” Exposure of laboratory rodents to a predator, such as a cat, elicits defensive behaviors even if they have never been exposed to cats before; therefore, such behaviors appear to be innate and not experiential, as Darwin posited. The *fear emotion might be the most crucial emotion* for survival via eliciting defensive responses, such as fighting, and thus has been preserved throughout evolution (Olsson and Phelps, 2007).

### Basic Emotions Manifest as Stereotypical Behavioral Phenotypes

Emotion is a kind of internal drive, which can exert a powerful effect on behavioral choice (Pereira and Murthy, 2017). Basic emotion related internal drive can induce behaviors that are supported by genetically hardwired neural circuits and are critical for animals’ survival. Several major categories of innate behavior, such as feeding, reproduction, aggression, and sleep, are observed across animal species. As hierarchical systems comprised of behavioral subprograms, innate behaviors are not only robust but can also fluctuate in intensity and adapt to an organism’s internal and external contexts (Kim et al., 2017). Fear is an important hereditary gift that aids survival by protecting against dangerous situations (Becker, 1997). All animals have specific behaviors to defend against predators, and Darwin found that “even insects express fear, anger, jealousy and love, by their stridulation” (Darwin, 1998). At stressful situation, emotion anger develops after fear emotion disappear, and manifests as fighting to cope with stressful situations, which can be easily observed in invertebrates. For example, the stings of bees, spiders, flies, ants, and scorpions are powerful tools to protect the organisms from predators. In addition, fighting also serves in the acquisition and defense of vital resources, such as food, shelter, or access to mates, such as what Sturtevant reported the fighting behavior in *Drosophila* (Chen et al., 2002). In stressful situations in which two males are courting the same female, he wrote “in such cases they (males) may be seen to spread their wings, run at each other, and apparently butt heads. One of them soon gives up and runs away. If the other then runs at him again within the next few minutes he usually makes off without showing fight” (Chen et al., 2002; Kim et al., 2018). Although it is difficult to determine whether fighting behaviors in insects are manifestations of an internal emotional state of anger like in humans, the function of fighting behaviors of invertebrates is very similar to the defensive behaviors of mammals; e.g., biting, clawing, hissing, and arching the back are fighting behaviors by which mammals threaten dangers away. Furthermore, like mammals, emotions in invertebrates are transient internal states, such that anger in insects manifests after fear to cope with the stressful situation (Zheng et al., 2016). It is necessary for survival even in *Drosophila*, and fight-or-flight behaviors can be easily seen in *Drosophila*, the flies are ready to fight with wings up for threat at a stressful situation, and after a series of stereotypical fighting behaviors one fly admits failure and fly away (Darwin, 1872).

### Basic Emotions Gain New Meanings Throughout Evolution

Despite the universal nature of basic emotions, new behaviors of emotions have emerged via evolution (Ekman, 1992b). Some emotion related behaviors might be learned, e.g., the stress-related emotions of fear and anger have also got new meanings over time. While the original meaning of fear entails life-threatening situations with fight or flight behaviors in response to predators, new meanings of fear are generalized to any situations that occur unexpectedly. In addition the original meaning of happiness is associated with pleasurable feelings and generates approach and consummator behaviors, eventually leading to behavioral reinforcement (Schultz et al., 1997), the new meaning of happiness also gained new meanings through evolution: whether the organism is able to cope successfully (Yurkovic et al., 2006).

Consistently, the neuromodulator dopamine (DA), is known to mediate unconditioned pleasure and reward from food, sex, and drugs, but recent findings suggest that DA is also involved in the anticipatory, preparatory, approach, and coping phases of reward behavior (Schultz et al., 1997; Sandoval and Seeley, 2017). This is consistent with Lazarus’s reappraisal theory about happiness and sadness, which posits that happy and sad emotions are related to the success or failure of coping with stressful situations, respectively (Lazarus, 1999). The happiness in insects parallels that of mammals in several aspects. In *Drosophila*, happy emotion is expressed as locomotive activity (Mansourian et al., 2016; Wang and Sokolowski, 2017), which also happens after successful coping. Depending on studies in *Drosophila*, Kim et al. (2018) proposed that winning is perceived as rewarding, while losing is aversive. Presentation of food promotes a state of elevated locomotive activity, which can be controlled by DA. Furthermore, during courtship, males extend their wings horizontally and vibrate them to generate a “song” that attracts females, and the optogenetic activation of specific brain interneurons that control the courtship song can lead to persistent singing for several minutes (Dickson, 2008; von Philipsborn et al., 2011; Inagaki et al., 2014). In contrast, *Drosophila* raise their wings vertically into the “wing-threat” position during antagonistic interactions with conspecific males, and after a series of stereotypical defensive behaviors, one fly lowers its wings to admit failure and retreats (Chen et al., 2002).

## Biological Aspects of Emotions

Given that basic emotions evolved to handle fundamental life tasks, it would follow that there must be biological patterns that drive each emotion. Ekman (1992b) proposed that basic emotions have many characteristics that distinguish one emotion from another, such as universal signals, distinctive physiology, and automatic appraisal influenced by both ontogenetic and phylogenetic past. Therefore, basic emotions not only provide information through behavioral expression to conspecifics, but specific biological changes prepare the organism to respond differently to various states of emotion. However, many studies using fMRI failed to get consistent results about specific neural basis for specific basic emotions (Lindquist et al., 2012), which lead to many psychologists try to give up the basic emotion theory (Lindquist et al., 2013; Barrett and Russell, 2015). Here we review basic emotions in *Drosophila*, whose brain structure are totally different from that of humans, but the neuromodulator are similar among all these animals. Numerous studies have pointed to an important role for neuromodulators (e.g., DA, 5-HT, and NE) in emotional process (Pereira and Murthy, 2017). Neuromodulators are believed to control the internal states related to emotions, mood, and affects, and exert critical influences on emotion related behaviors (Watanabe et al., 2017). Therefore, we propose that *emotion is an internal state, whose neural substrate is the neuromodulator* (Kim et al., 2018). For example, norepinephrine is a potent modulator of brain-wide states such as arousal (Suver et al., 2012; Pereira and Murthy, 2017).

### Neural Basis for Basic Emotions

**FIGURE 2:**
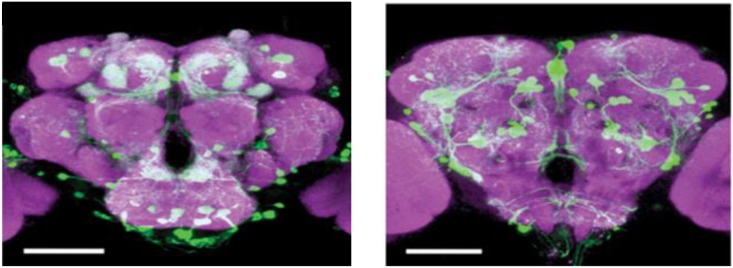
DA cells in the *Drosophila* brain. An anterior view of DA neurons in the *Drosophila* brain. Labeling of DA cells and processes was achieved by a tyrosine-hydroxylase enhancer trap driving expression of green fluorescent protein (White et al., 2010), Scale bar, 100 μm (courtesy of Dr. Frank Hirth, King’s College London).

Insects lack homologs of vertebrate forebrain structures involved in emotional processing, such as the nucleus accumbens (NAc), prefrontal cortex (PFC), amygdala, and hippocampus; however, insects have evolved structures such as the mushroom body and central complex that show many functional and anatomical similarities to the mammalian structures that mediate basic emotions (Lin A.C. et al., 2014; Lin S. et al., 2014). The monoamine system in invertebrates (Klemm et al., 1985) and non-mammalian vertebrates (Perry and Capaldo, 2011) has been implicated in stress by numerous studies. Comparative anatomical studies of the neurons releasing OA, DA, and 5-HT have shown striking similarities among different insect species (Konings et al., 1988; Dacks et al., 2005) and point to a stereotypic pattern of neurons that are widely distributed in the central nervous system (CNS) of flies. In *Drosophila* brain, there are about 100 octopaminergic neurons (Sinakevitch et al., 2005), which can be divided into two kinds of neurons: interneurons and efferent neurons. The efferent neurons project diffusely to almost all the neuropil structure in the brain (Sinakevitch et al., 2005). Like the NE in vertebrates, the counterpart of OA, some octopaminergic neurons have been prove to be implicated in aggression (Ramirez and Pearson, 1991; Schroter et al., 2007; Andrews et al., 2014). Similarly, there are approximately 40 5-HT neurons in the *Drosophila* brain, whose innervations are spread around the feeding apparatus and also the ring gland (Sitaraman et al., 2008). On the contrary, the dopaminergic neurons project to the mushroom bodies to control behaviors (Figure 2; Zhang et al., 2007). Similar to 5-HT, DA neurons are interneurons, which connect different regions of neuropil. DA neurons show hydroxylase-immunoreactivity, and locate in the central body, anterior protocerebrum and other scattered region (Han et al., 1996). A cluster of approximately 130 dopaminergic neurons that innervate the horizontal lobes of the mushroom body was implicated sugar reward (Burke et al., 2012; Liu et al., 2012; Figure 2).

### Norepinephrine – Stress

The norepinephrine system of mammals influences various aspects of the animal’s life, including *Drosophila*, and they can modulate a variety of physiological processes and behaviors in response to stress. Stressful stimuli induce a metabolic and behavioral adaptation, leading to enhanced energy supply, increased muscle performance, increased sensory perception and a matched behavior. This so-called “fight or flight” response can be seen in both vertebrates and invertebrates. Cannon said: “The highest level of modulatory monoamine input occurs during “fight or flight” behavioral situations.”

Fighting is a primitive behavior and is regarded as one of the fundamental instincts behind innate animal behaviors by Konrad Lorenz and Nikolaas Tinbergen, the founders of modern neuroethology (Asahina, 2017). Fighting in *Drosophila* offers a unique opportunity for studying basic emotions because of its stereotypical actions and its easily identified genetic resources. The fighting behavior in *Drosophila* is activated by pheromones and internal states, such as hunger. *Drosophila* recognizes the sex, species and even the food quality by chemosensation (Wang et al., 2011; Clowney et al., 2015). For example, 11-cis-vaccenyl acetate is a specific male odor chemical, and has been shown to be involved in male-male fighting (Wang and Anderson, 2010; Liu et al., 2011). The yeast smell which might signify food quality can induce female aggression (Ueda et al., 2002; Nelson and Trainor, 2007; Asahina et al., 2014). Even though these cues can induce fighting, the internal status of the animals can affect the fighting too, for example, prior defeat can reduce fighting behavior in many animal species (Penn et al., 2010; Hammels et al., 2015). In addition to past fighting experience, fighting behavior can also be influenced by the outcome of other behaviors, including recent mating success, hunger levels, and sleepiness. For example, recent mating experience can increase fighting tendency (Yuan et al., 2014), whereas sleep deprivation can decrease the frequency of fighting (Kayser et al., 2015). Lorenz already found the importance of internal states in fighting and suggested that fighting threshold can be lowered by an aggressive drive (anger) (Asahina, 2017).

It might be due the fact that increase octopamine can modulate the flight-or-fight response by affecting chemosensation responses (Stevenson et al., 2005; Ramdya et al., 2015). Amines like dopamine, tyramine, octopamine, and serotonin, were among the first molecules to be implicated in *Drosophila* aggression, in large part because previous studies in lobsters and crickets suggested strongly that monoamine neuromodulators affect aggression (Asahina, 2017). It has been well-known that the function of NE system is to induce fight-or-flight behaviors and serve to help the organism cope with dangerous environment (Zheng et al., 2016). Following exposure to a threat, NE is released from sympathetic nervous system to the blood, and directly affects the heart rate, and triggers the glucose release. NE is also released from locus coeruleus in the central nervous system. LC neurons project very profusely to most regions of the brain, and can influence the whole brain (Yurkovic et al., 2006). Octopamine (OA) in insects is a neuroactive substance that has a chemical structure that closely resembles NE and can function as a neurohormone for basic emotion (Arnott and Elwood, 2009; Lovheim, 2012; Asahina, 2017). The activity of octopaminergic neurons was initially discovered in octopus’s salivary glands, and has since been shown to mediate stress-response in many invertebrate (Sinakevitch et al., 2005; Stevenson et al., 2005; Burke et al., 2012). In addition, OA has been shown to be involved in fighting and also in elevated flight in crickets (Gammie et al., 2005; Stevenson et al., 2005; Asahina, 2017). In male *Drosophila*, octopamine is necessary to maintain levels of aggression (Zhou et al., 2008). A null mutation in the gene that encodes tyramine-β-hydroxylase (TβH), which catalyzes the OA synthesis, can significantly reduce fighting and aggression (Andrews et al., 2014; Asahina, 2017), which suggest its role in maintenance of fighting (Andrews et al., 2014). Even though activation of octopaminergic neurons or exogenous administration of octopamine, the invertebrate counterpart of noradrenaline can activate aggression (Schretter et al., 2018), whether an octopaminergic signal is sufficient to elevate levels of aggression remains unclear. For example, even though OA plays a role in fighting or aggression, artificially administering OA or an OA-agonist results in mixed effects on aggression (Hoyer et al., 2008; Kayser et al., 2015). In addition, overexpression of TβH does not increase fighting (Hoyer et al., 2008), instead it induced conflicting outcomes (Certel et al., 2010; Kayser et al., 2015). This might be due the fact that OA and NE systems are the substrates for both fear and anger, or fight and flight behaviors. OA-dependent modulation of organs and tissues is mainly elicited through muscle action, especially in terms of its impact on the “fight or flight” response. Flight is also an important and critical behavior in flying insects. Here the “flight” means flying away, instead of the normal “fly.” Neuromodulation of insect “flight” has thus far been attributed primarily to biogenic amines (Brembs et al., 2007). However, there are few studies in *Drosophila* about the “flight” behavior (Shen, 2012). However, studies about the innervation pattern in the periphery also support the idea that the OA/TA system is crucial for insects to switch from a dormant to an excited state, by a positive modulation of muscle activity, heart rate, and energy supply, and a simultaneous negative modulation of physiological processes like sleep (Agrawal and Hasan, 2015; Pathak et al., 2105; Pauls et al., 2018).

#### Dopamine – Reward

Octopamine was historically considered to be the signal for reward in insects, only recently has dopamine been linked to motivated behavior and rewarding reinforcement in fruit flies (Burke et al., 2012). The hedonic hypothesis of DA was first proposed by Wise and posits that DA in the brain plays a critical role in the subjective pleasure associated with positive reward and that a reduction in DA results in a loss of pleasure (Bozarth and Wise, 1980; Gu et al., 2016). Afterward, many studies have proved the role of DA in reward signaling (Dougherty et al., 1999; Matthews et al., 2016), notably many pharmacological and behavioral studies have confirmed the important role of medial prefrontal DA system in reward behaviors (Keleman et al., 2012). In addition, studies of the mechanisms of some drugs of abuse also support the role of DA in reward system by increasing presynaptic release of DA and inhibiting DA reuptake, such as cocaine and amphetamine (Narita et al., 2008; Del Campo et al., 2011). In contrast, decreased striatum DA responses were reported in detoxified cocaine abusers. Together, these studies suggest an involvement of DA neurotransmission in the reward process (Matsumoto et al., 2016), and this hypothesis has significantly impacted theories of drug addiction, and motivation since it was first introduced.

However, recent DA studies have opened this theory to reexamination (Berridge and Kringelbach, 2015). The incentive salience hypothesis has recently been accepted, which suggests that the major function of DA is anticipatory, preparatory, approach, instead of unconditioned pleasures from food, sex, or drugs (Schultz et al., 1997; Sandoval and Seeley, 2017). Therefore, Hailan Hu proposed that happiness = reward minus predicted reward (Dickson, 2008; Hu, 2016; Matsumoto et al., 2016), which means surprise can enhance happiness (Lazarus, 1999). Similar in *Drosophila*, OA (surprise) was thought to be involved in reward in insects (Burke et al., 2012), including *Drosophila* (Zhang et al., 2007; Liu et al., 2017). It is found that OA can trigger activation of dopaminergic neurons (Burke et al., 2012; Kahnt et al., 2012). Analysis of the β-adrenergic-like OCTβ_2_R receptor suggest that this OA-dependent reinforcement requires an interaction with dopaminergic neurons that control appetitive motivation (Burke et al., 2012; Kahnt et al., 2012). These evidence suggests clear roles for DA in reward-related processes in invertebrates (Ma et al., 2016), including motivation behavior and nutritional valuation of reward (Arnott and Elwood, 2009).

Historically, DA has been suggested to be most prominently associated with reward and punishment (Liu et al., 2012), recent findings from *Drosophila* confirmed all these functions, as well as additional roles in the interplay between external sensation and internal states (Burke et al., 2012; Kaun and Rothenfluh, 2017). It is believed that drug addiction is due to the mechanism of drugs of abuse “hijacking” the dopaminergic “reward” circuit and thus these artificial rewards reinforce associated behaviors. Recent complicating dopaminergic involvement in addiction has been proved to modulate internal state of the animals (Kim et al., 2017). Many drugs of abuse, such as alcohol stimulate locomotion, can induce *Drosophila* hyper-locomotion, and PPM3 DA neurons in mushroom body has been suggested to be involved in changing the activity and arousal states of the flies (Kim et al., 2017). Different kinds of drugs with different stimulation salience can induce different dopaminergic activity in distinct DA neurons in the mushroom body.

#### Serotonin – Punishment

Although DA release mediates reward, inhibition of DA should induce punishment. However, evolution created a separate process for punishment, and while it may seem redundant, 5-HT has been known to play a critical role in punishment. It is found that approximately 90% of 5-HT is secreted by gut chromaffin cells in response to noxious food, thus inducing vomiting or diarrhea (Beyeler, 2016; Zheng et al., 2016). Some plants exploit this function of 5-HT by expediting passage of seed through the digestive system. In addition, some animals such as scorpion and wasp stings also use 5-HT to induce pain (Gu et al., 2016). Aversion is separated from reward in the evolutionarily lower animals (Beyeler, 2016). The nematode, *Caenorhabditis elegans*, can also be infected by pathogenic bacteria, even though it feeds on bacteria. Indeed, exposure to pathogenic bacteria can enhance the 5-HT release, which can induce negative reinforcing reflex in *C. elegans*. However, the function of 5-HT gets more complicated during evolution, for example, it is found that some 5-HT neurons are involved in the positive rewarding process (Liu et al., 2012; Ries et al., 2017). It is found that some 5-HT neurons in dorsal raphe nucleus fire consistently during acquisition of a variety of rewards, including sex (Isosaka et al., 2015). Some studies reported a correlation between 5-HT level and positive emotions in rodents (Kvajo, 2016). Therefore, it might be too complicated to differentiate the functions of DA and 5-HT in such highly evolutionary creatures such as mammals and human beings. As such, *Drosophila* offered a very good model to test the functions of DA or 5-HT in positive or aversive reinforcement (Zhang et al., 2016).

The emotions are internal states evoked by sensing for bodily instinctual needs, and underlined by neuromodulator release, and will promote some behaviors. In mammals, these neuromodulators are primarily monoamines, which are highly interconnected with a network of modulators and transmitters important for complex behaviors. Neuropeptides and monoamines are very often expressed in combination with each other and with neurotransmitters. Possible interactions between neuromodulators and neurotransmitters are interesting and important issues. A dozen neuromodulator systems in *Drosophila* have been implicated to date in basic emotion, including serotonin, octopamine, acetylcholine, glutamate, and GABA, and many neuropeptides, and many of these neuromodulator systems appear to be functionally conserved throughout evolution, including orthodox for mammalian peptidergic signals tachykinin, cholecystokinin, neuropeptide Y, Neuromedin U and insulin (Pool and Scott, 2014). However, work in *Drosophila* suggests that the rewarding drives are majorly gated through DA neurons, or DA plays a central role in creating the motivational drive underlying many behaviors (Kim et al., 2017). In male flies, dopaminergic neurons of the ventral nerve cord promote persistent copulation, and a subset of dopaminergic neurons innervating the mushroom body is required for persistent courtship. Similarly, even neuropeptide are shown to affect *Drosophila* aggression, such as neuropeptide F, a functional homolog of vertebrate neuropeptide Y. However, NPF-expressing neurons seem to play a more general role in modulating male behavioral patterns when potential competitors are present (Asahina, 2017). Unlike octopamine, which is clearly an important neuromodulator for aggression, NPF has also been regarded as neuromodulator of feeding behavior across animal species, and activation of NPF signaling mimics the hunger states. In addition, there is no specific population of NPF expression neurons, unlike the neurons for three monoamines (Asahina, 2017; Ryglewski et al., 2017).

**FIGURE 3:**
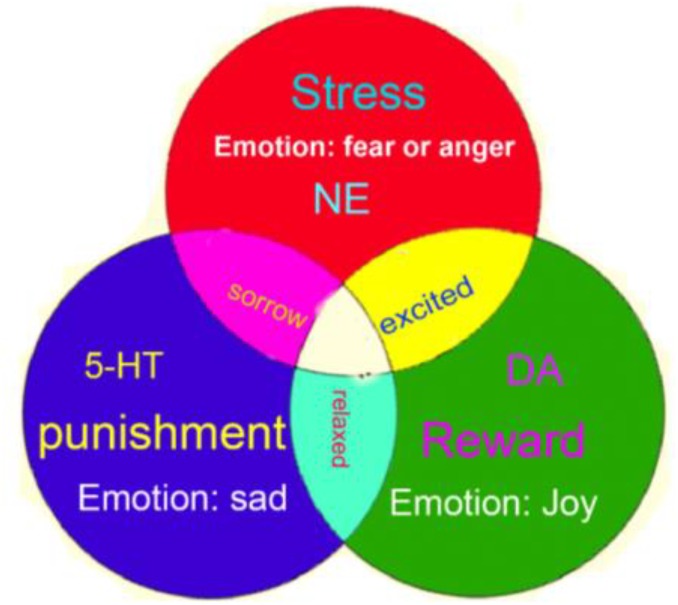
Three-primary-color model of core affects. The three monoamine neuromodulators are the substrates for three core affects (norepinephrine-stress, dopamine-reward, 5-HT-punishment). And the three core affects constitute the basic emotions: stress-fear and anger, reward-happiness or joy, punishment-sadness or disgust.

Therefore, we propose that the monoamine neuromodulators underlie the three core affects of basic emotions; specifically, NE is related to the fight-or-flight responses at stressful events, DA is involved in reward, and 5-HT is related to punishment (Figure 3). Consistently, a paper in PNAS proposed that the use of *Drosophila* as a model for circuit dissection of internal states can promote behavioral changes associated with winning or losing after coping: Winning is perceived as rewarding, while losing is aversive (Kim et al., 2018).

## Conclusion

In this review, we propose a framework for the evolutionary study of emotions based on behavioral observations of *Drosophila*. From analysis of molecules and neural systems to observational study of behaviors and social functions, the *Drosophila* model is a powerful tool to understand the evolutionary origin and neurobiological underpinnings of emotions (Anderson, 2016; Kim et al., 2018). The brain structure of *Drosophila* are totally different from that of humans, but they have similar neuromodulators and innate states (Kim et al., 2018). Numerous studies have pointed to an important role for neuromodulators (e.g., DA, 5-HT, and NE) in the emotional process (Pereira and Murthy, 2017). Neuromodulators are believed to control the internal states related to emotions, mood, and affects, and exert critical influences on emotion related behaviors (Watanabe et al., 2017).

### Emotion Is an Innate State, Whose Neural Substrate Is the Neuromodulator Release

We demonstrated that basic emotions are primitive, internal states that have gained new meanings and new external behavioral expression via evolution in order to meet organisms’ biological, social, and functional needs (Ekman, 1992b; Anderson and Adolphs, 2014). Reward, punishment, and stress are the three most primitive features of the four basic emotions (happiness, sadness, fear, anger) and are driven by the three monoamine neuromodulators (DA-reward, 5-HT-punishment, NE-stress). These three monoamines are not only the substrates for the four basic emotions, but we posit that these monoamines combine in varying degrees to ultimately create various higher order emotions, much like the way different colors can be created from the three primary colors; we call this the “Three Primary Color Model of Basic Emotions.”

This paper establishes a new theory of emotion. A scientific theory in psychology, similar to those in physics, is its elegance and beauty in describing a vast area of knowledge from few basic principles, or use few fundamental principles to describe a vast area of knowledge (Perlovsky, 2016b). Traditional psychology is a “soft” science that does not develop models of the mind based on few principles, describing vast areas of knowledge, and making experimentally verifiable predictions (Perlovsky, 2016b). Here we introduced the very simple model for basic emotions, a very simple theory about emotions. It might be an oversimplification to categorize monoamine simply as an aggression-promoting neuromodulator, but we hope our hypothesis can help understand the basic emotion theory. For validation, detailed studies of the specific behavioral expressions of states of relative excess or deficit of the neurotransmitters 5-HT, NE, and DA may offer confirmatory observations supporting this model of emotions.

## Author Contributions

FW and SG designed the manuscript. FW, JB, and JH wrote the manuscript. NP revised the manuscript.

## Conflict of Interest Statement

The authors declare that the research was conducted in the absence of any commercial or financial relationships that could be construed as a potential conflict of interest.
